# Adducin family proteins possess different nuclear export potentials

**DOI:** 10.1186/s12929-017-0333-0

**Published:** 2017-05-10

**Authors:** Chia-Mei Liu, Wen-Hsin Hsu, Wan-Yi Lin, Hong-Chen Chen

**Affiliations:** 1 Department of Life Sciences, National Chung Hsing University, Taichung, Taiwan; 2 Program in Tissue Engineering and Regenerative Medicine, National Chung Hsing University, Taichung, Taiwan; 3 Institue of Biomedical Sciences, National Chung Hsing University, Taichung, Taiwan; 4 Rong-Hsing Research Center for Translational Medicine, National Chung Hsing University, Taichung, Taiwan; 5 Institute of Biochemistry and Molecular Biology, National Yang Ming University, No. 155, Sec. 2, Li-Nong St, Taipei, 11221 Taiwan

**Keywords:** Adducin, Nuclear localization, Nuclear export, RNA polymerase, ZNF331

## Abstract

**Background:**

The adducin (ADD) family proteins, namely ADD1, ADD2, and ADD3, are actin-binding proteins that play important roles in the stabilization of membrane cytoskeleton and cell-cell junctions. All the ADD proteins contain a highly conserved bipartite nuclear localization signal (NLS) at the carboxyl termini, but only ADD1 can localize to the nucleus. The reason for this discrepancy is not clear.

**Methods:**

To avoid the potential effect of cell-cell junctions on the distribution of ADD proteins, HA epitope-tagged ADD proteins and mutants were transiently expressed in NIH3T3 fibroblasts and their distribution in the cytoplasm and nucleus was examined by immunofluorescence staining. Several nuclear proteins were identified to interact with ADD1 by mass spectrometry, which were further verified by co-immunoprecipitation.

**Results:**

In this study, we found that ADD1 was detectable both in the cytoplasm and nucleus, whereas ADD2 and ADD3 were detected only in the cytoplasm. However, ADD2 and ADD3 were partially (~40%) sequestered in the nucleus by leptomycin B, a CRM1/exportin1 inhibitor. Upon the removal of leptomycin B, ADD2 and ADD3 re-distributed to the cytoplasm. These results indicate that ADD2 and ADD3 possess functional NLS and are quickly transported to the cytoplasm upon entering the nucleus. Indeed, we found that ADD2 and ADD3 possess much higher potential to counteract the activity of the NLS derived from Simian virus 40 large T-antigen than ADD1. All the ADD proteins appear to contain multiple nuclear export signals mainly in their head and neck domains. However, except for the leucine-rich motif (^377^FEALMRMLDWLGYRT^391^) in the neck domain of ADD1, no other classic nuclear export signal was identified in the ADD proteins. In addition, the nuclear retention of ADD1 facilitates its interaction with RNA polymerase II and zinc-finger protein 331.

**Conclusions:**

Our results suggest that ADD2 and ADD3 possess functional NLS and shuttle between the cytoplasm and nucleus. The discrepancy in the subcellular localization of the ADD isoforms arises due to their different nuclear export capabilities. In addition, the interaction of ADD1 with RNA polymerase II and zinc-finger protein 331 implicates a potential role for ADD1 in the regulation of transcription.

## Background

The proteins in the karyopherin-β family, which are also known as importins and exportins, mediate the nucleocytoplasmic shuttling of most macromolecules [1, 2]. These karyopherins recognize and bind to specific signal sequences that are present on the cargo molecules and transport them into and out of the nucleus through nuclear pore complexes. Nuclear localization signals (NLSs) recognized by importins direct proteins into the nucleus, whereas nuclear export signals (NESs) recognized by exportins direct the export of proteins from the nucleus to the cytoplasm. Typically, nuclear proteins possess a short positively charged NLS, such as the PKKKRKV sequence of the Simian virus 40 large T antigen (SV40 T-Ag) [3, 4]. The NES within the HIV-1 Rev protein, the leucine-rich sequence LPPLERLTL, was the first NES to be identified [5, 6]. So far, two classes of NLSs, known as the classic NLS and the PY-NLS, and one class of NES, known as the leucine-rich or classic NES, have been characterized [7, 8].

NESs have been identified in more than 200 proteins, and many of those do not necessarily contain leucine residues but rather have generally hydrophobic patterns [9]. They conform loosely to the widely used traditional consensus of Φ1-*X*_2,3_-Φ2-*X*_2,3_-Φ3-X_1_-Φ4 pattern, where "Φ" represents Leu, Val, Ile, Phe, or Met and "X" can be any amino acid [10]. However, because large portions of the consensus patterns essentially describe the amphipathic helix, which is found in most proteins, there exist many false-positive sequences that conform to the NES consensus but do not have the capability for nuclear export. Therefore, successful identification or prediction of NESs has been hindered by the very broad traditional consensus sequence. In most cases, NESs have been experimentally identified and reported on an individual basis.

Leptomycin B (LMB) is an anti-fungal antibiotic that acts as an inhibitor of nuclear export by forming a covalent complex with the sulfhydryl group of a conserved cysteine residue in CRM1 (chromosomal maintenance 1, also known as exportin1), thereby inhibiting CRM1 interaction with the NES of the targeted export proteins [11]. The ability of LMB to inhibit nuclear export has made it a useful tool in the study of the subcellular localization of many regulatory proteins. LMB blocks the nuclear export of many proteins, such as HIV-1 Rev [12], MAPK/ERK [13], and NFκB/IκB [14].

Adducin (ADD) proteins are actin-binding proteins that are mainly localized at actin-spectrin junctions [15]. The ADD family consists of three closely related genes: ADD1 (α isoform) and ADD3 (γ isoform) are found in most tissues, whereas ADD2 (β isoform) is abundant in erythrocytes and the brain [16–18]. ADD proteins are known to be important for the stabilization of the membrane cortical skeleton [15, 19] and cell-cell contacts [20, 21], cell migration [22], and the maintenance of the structure and function of neuronal axons [23]. All the ADD isoforms (ADDs) are similar in their amino acid sequences and domain structures, consisting of an amino-terminal head domain, a neck domain, and a carboxyl-terminal tail domain with a highly conserved bipartite NLS [24, 25]. It has been shown that ADD1 is translocated to the nucleus upon loss of cell-cell adhesion [21]. In addition, ADD2 was found in nucleus in response to pleiotrophin [26], a secreted heparin-binding cytokine [27]. However, the nuclear function of ADDs and the mechanisms for their nucleocytoplasmic shuttling remain elusive. In this study, we demonstrate that the discrepancy in the subcellular localization of the ADD isoforms arises from the differences in their nuclear export capability. In addition, the nuclear retention of ADD1 facilitates its interaction with RNA polymerase II and zinc-finger protein 331 (ZNF331), which implicates a role for ADD1 in regulating nuclear activities.

## Methods

### Plasmids

For the HA epitope tagged-ADD (HA-ADD), the cDNAs of ADD1, ADD2, and ADD3 were cloned into the BamHI and EcoRI sites of the pKH3 plasmid. The plasmid pCMV-3Tag-3A-ADD1 for the FLAG epitope-tagged ADD1 (FLAG-ADD1) was constructed in our laboratory and described previously [28]. The plasmid pEGFP-C1-NLS^SV40^ was described previously [29]. For the GFP-C1-NLS^SV40^-ADD1, ADD1 cDNA was cloned into the KpnI and BamHI sites of the pEGFP-C1-NLS^SV40^ plasmid. For the GFP-C1-NLS^SV40^-ADD2, ADD2 cDNA was cloned into the EcoRI and BamHI sites of the pEGFP-C1-NLS^SV40^ plasmid. For the GFP-C1-NLS^SV40^-ADD3, ADD3 cDNA was cloned into the BglII and EcoRI sites of the pEGFP-C1-NLS^SV40^ plasmid.

### Cell culture and transfections

NIH3T3, HEK293, A431, and HeLa cells were maintained in DMEM supplemented with 10% fetal bovine serum and cultured at 37 °C in a humidified atmosphere of 5% CO_2_ and 95% air. For transient transfections, the cells (2 × 10^5^) were seeded in 6-cm culture dishes. After 24 h, the cells were incubated with the mixture of plasmid DNA (2 μg) and Lipofectamine (6 μl) (Invitrogen) for 6 h and allowed to grow for another 24 h. To inhibit nuclear export, the cells were treated with 20 nM LMB (Enzo Life Sciences) for 3 h. In some cases, LMB was removed by extensive washing and the cells were further incubated for 18 h prior to fixation for immunofluorescence staining.

### Lentiviral production

The lentiviral expression system and the pLAS3w.Pneo plasmid were obtained from the National RNAi Core Facility (Academia Sinica, Taiwan). To produce the lentiviral particles, HEK293T cells were transfected with 2.25 μg pCMV-∆R8.91, 0.25 μg pMD.G, and 2.5 μg pLAS3w.Pneo-FLAG-ADD1 using Lipofectamine. After three days, the medium containing the lentiviral particles was collected and stored at -80 °C. To generate HeLa or A431 cells stably expressing FLAG-ADD1, the cells were infected with lentivirus encoding FLAG-ADD1 for 24 h and were subsequently selected in the medium containing 0.5 mg/ml G418.

### Immunoblotting and immunoprecipitation

To prepare cell lysates, the cells were lysed in 1% NP-40 lysis buffer (1% NP-40, 20 mM Tris-HCl, pH 8.0, 137 mM NaCl, 10% glycerol, and 1 mM Na_3_VO_4_) containing protease inhibitors (phenylmethylsulfonyl fluoride, aprotinin, and leupeptin). The lysates were incubated with anti-HA, anti-CRM1 (Santa Cruz Biotechnology), anti-exportin5 (Santa Cruz Biotechnology), anti-RNA polymerase II (Santa Cruz Biotechnology), or anti-ADD1(Santa Cruz Biotechnology) antibodies for 1.5 h at 4 °C, and the immunocomplexes were precipitated by protein A–Sepharose beads (GE Healthcare Life Sciences). For the mouse antibody, protein A–Sepharose beads were coupled with 0.5 μg rabbit anti–mouse IgG before use. The beads were washed three times with 1% NP-40 lysis buffer boiled for 3 min in SDS sample buffer, subjected to SDS-polyacrylamide gel electrophoresis, and transferred to nitrocellulose membranes. Immunoblotting was performed with the indicated antibodies using the Western chemiluminescent HRP substrate for the detection. Chemiluminescent signals were detected and quantified by the Fuji LAS-4000 mini luminescence imaging system.

### Immunofluorescence staining

**Fig. 1:**
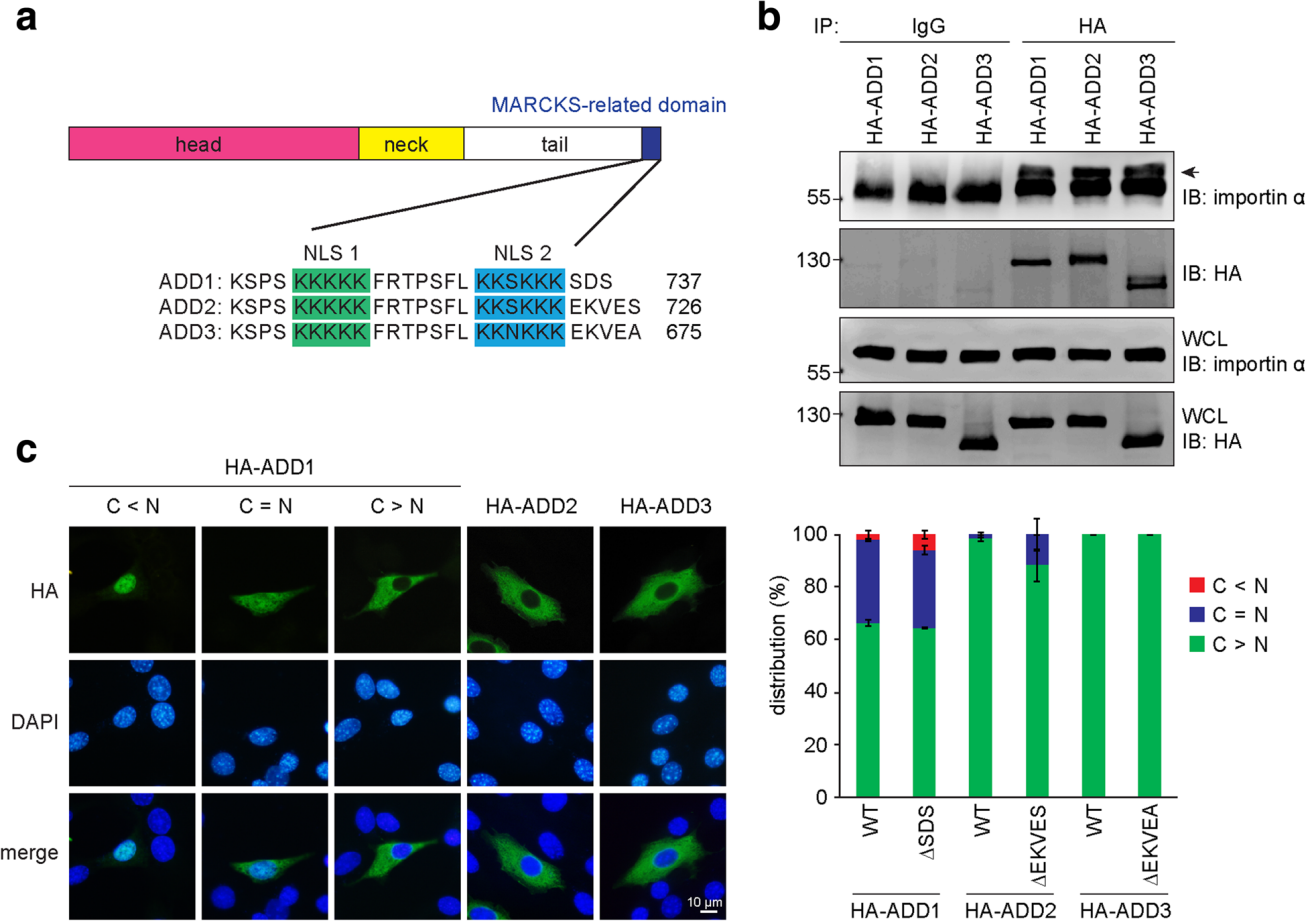
ADD1 was detected in the nucleus and cytoplasm, whereas ADD2 and ADD3 were detected only in the cytoplasm. **a** Schematic representation of the NLS motif in ADD1, ADD2, and ADD3. **b** HA-ADD1, HA-ADD2, and HA-ADD3 were transiently expressed in NIH3T3 cells. The cells were lysed, and the interaction of HA-ADD proteins with importin α was analyzed by co-immunoprecipitation. The HA-ADD proteins were immunoprecipitated (IP) with anti-HA antibody or pre-immune serum (IgG) as the control. The immunocomplexes were washed and analyzed by immunoblotting (IB) with anti-importin α or anti-HA. An equal amount of whole cell lysates (WCL) was analyzed by immunoblotting to verify the levels of importin α and HA-ADD proteins. **c** HA-ADD1, HA-ADD2, and HA-ADD3 and their respective mutants with the deletion of residues adjacent to the NLS were transiently expressed in NIH3T3 cells. The cells were fixed and stained for the nuclei and HA-ADD proteins. The representative images are shown. Scale bar, 10 μm. The distribution of the HA-ADD proteins in the nucleus and cytoplasm was measured (*n* > 800). The values (mean ± SD) are from three experiments. *N* > C, distributed mainly in the nucleus; *N* < C, distributed mainly in the cytoplasm; *N* = C, distributed equally in the nucleus and cytoplasm

**Fig. 2:**
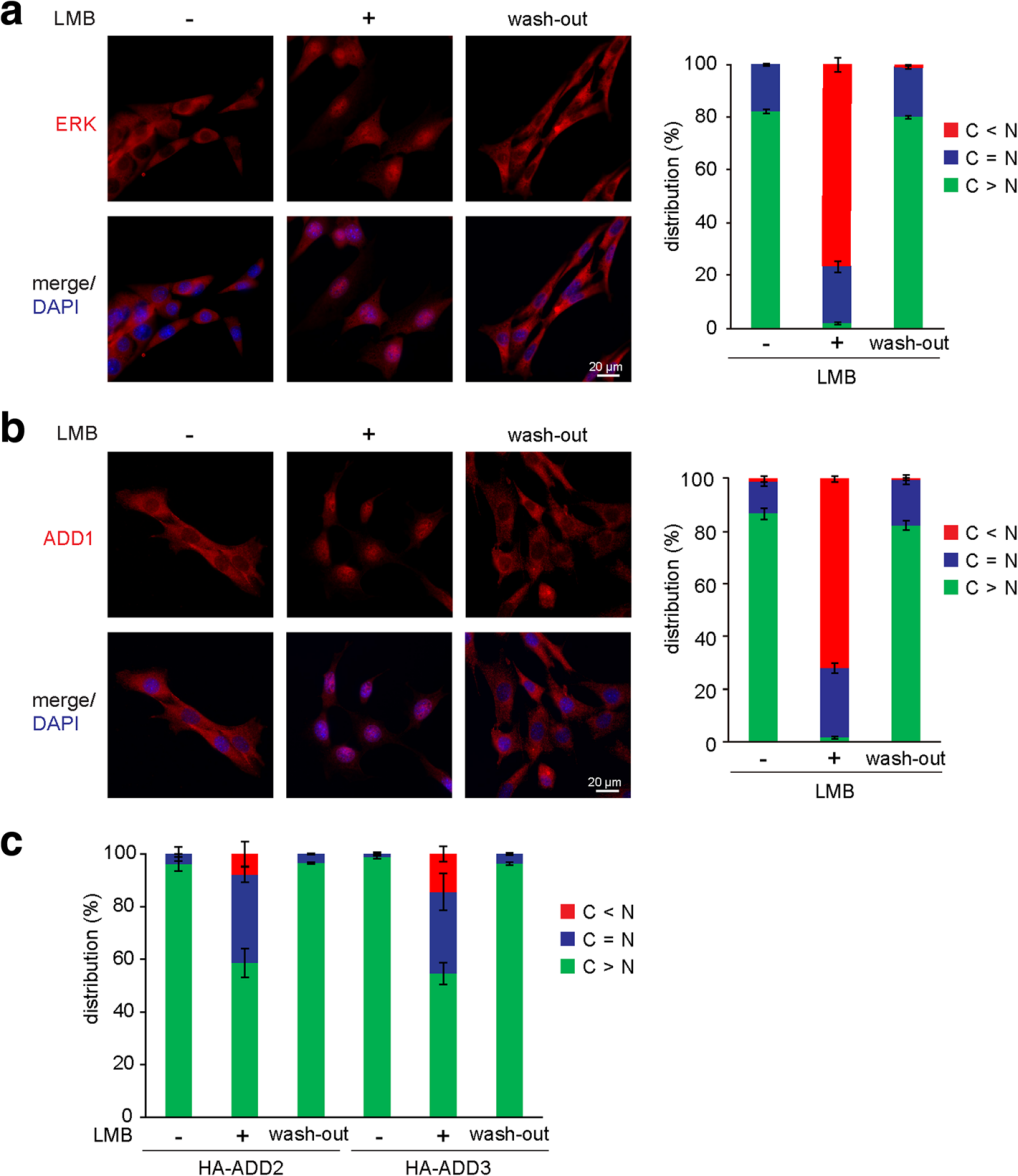
ADD1, ADD2, and ADD3 can be sequestered in the nucleus by LMB. **a** NIH3T3 cells were treated with (+) or without (-) 20 nM LMB for 3 h, followed by extensive washing to remove LMB (wash-out) before fixation. The cells were fixed and stained for the ERK protein and nuclei. The representative images are shown. The distribution of ERK in the nucleus and cytoplasm was measured (*n* > 600). The values (mean ± SD) are from three experiments. **b** NIH3T3 cells were treated with (+) or without (-) 20 nM LMB for 3 h, followed by extensive washing to remove LMB (wash-out) before fixation. The cells were fixed and stained for the ADD1 and nuclei. The representative images are shown. The distribution of ADD1 in the nucleus and cytoplasm was measured (*n* > 600). The values (mean ± SD) are from three experiments. **c** NIH3T3 cells transiently expressing HA-ADD2 and HA-ADD3 were treated with (+) or without (-) 20 nM LMB for 3 h, followed by extensive washing to remove LMB before fixation. The cells were fixed and stained for the HA-ADD and nuclei. The distribution of the HA-ADD proteins in the nucleus and cytoplasm was measured (*n* > 600). The values (mean ± SD) are from three experiments

**Fig. 3:**
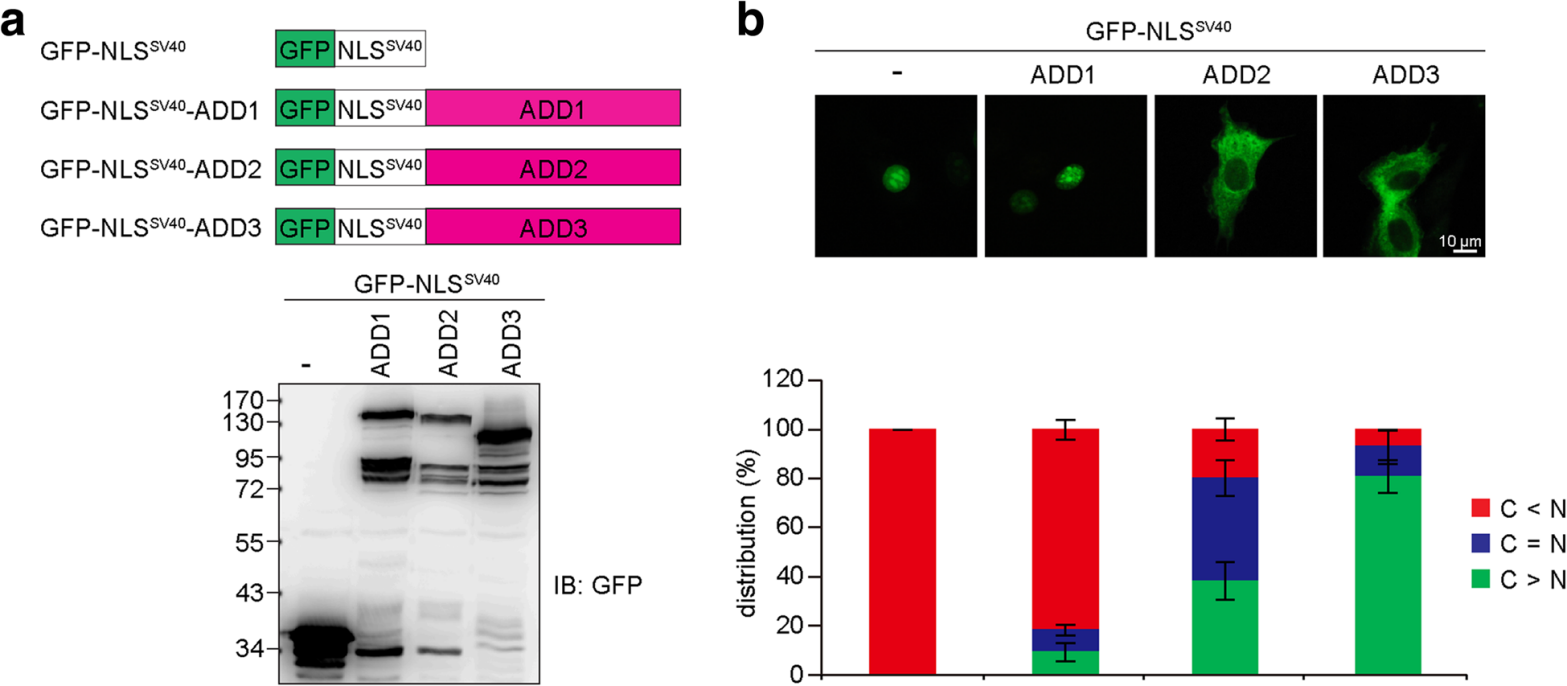
ADD2 and ADD3 counteracted the effect of the NLS derived from SV40 T-Ag. **a** The ADD isoforms were fused to GFP and the NLS derived from SV40 T-Ag. The fusion proteins (GFP-NLS^SV40^, GFP-NLS^SV40^-ADD1, GFP-NLS^SV40^-ADD2, and GFP-NLS^SV40^-ADD3) were transiently expressed in HEK293 cells and their expression was analyzed by immunoblotting with anti-GFP antibody. **b** The constructs described in panel a were transiently expressed in NIH3T3 cells and their distribution in the nucleus and cytoplasm was measured (*n* > 600). The values (mean ± SD) are from three experiments

Cells were fixed with 3% paraformaldehyde in 90% methanol at -20 °C for 30 min and permeabilized with 0.1% Triton X-100 for 10 min at room temperature. The fixed cells were stained with primary antibodies at room temperature for 2 h followed by incubation with Alexa Fluor 488- or 546-conjugated secondary antibodies (Invitrogen) for 2 h. The primary antibodies used for the immunofluorescence staining in this study were mouse anti-HA (16B12; 1:100) (Covance), mouse anti-FLAG (M2; 1:500) (Sigma-Aldrich), mouse anti-ZNF331 (1:50) (Abcam), rabbit anti-FLAG (1:500) (Sigma-Aldrich), rabbit anti-pol II (N-20; 1:50) (Santa Cruz Biotechnology), rabbit anti-ERK (sc-94; 1:200) (Santa Cruz Biotechnology) and rabbit anti-ADD1 (H-100; 1:100). Coverslips were mounted on the slides with mounting medium (Southern Biotech). The images in Figs. 1c and 3b were acquired with a LEICA epifluorescence microscope. The images in Figs. 2a, b, 7b, and d were acquired on an upright microscope (Axio imager. M2; Carl Zeiss) equipped with a 40× oil objectives (NA 1.3) and a camera (ORCA-Flash4.0 V2; Hamamatsu). The images were cropped with Photoshop CS6 (Adobe) and assembled with Illustrator CS6 (Adobe).

## Results

### ADD1 is detectable both in the nucleus and cytoplasm, whereas ADD2 and ADD3 are detected only in the cytoplasm

All the members of the ADD family contain a bipartite NLS motif in their tail domains (Fig. 1a) and are capable of interacting with importin α (Fig. 1b). However, upon transiently expression in the 3 T3 fibroblasts, equal distribution of HA-ADD1 in the nucleus and cytoplasm was detected in approximately 30% of the cells, whereas HA-ADD2 and HA-ADD3 were detected only in the cytoplasm (Fig. 1c). Deletion of the residues adjacent to the COOH-terminus of the bipartite NLS motif did not affect the distribution of the ADD proteins (Fig. 1c), indicating that the discrepancy in the subcellular distribution of the ADD proteins does not arise due to the residues adjacent to the NLS.

### The cytoplasmic distribution of ADD2 and ADD3 is the result of efficient nuclear export

To examine whether ADD2 and ADD3 could be translocated into the nucleus after protein translation in the cytoplasm, leptomycin B (LMB), an inhibitor for CRM1/exportin1 [11], was used in the experiments. LMB has been shown to efficiently block the nuclear export of MAPK/ERK [13], which serves as the control for ADDs. We found that LMB at the concentration of 20 nM, which is sufficient to sequester most (>80%) ERKs and ADD1 in the nucleus (Fig. 2a and b), partially (40%) sequestered ADD2 and ADD3 in the nucleus (Fig. 2c). Importantly, the removal of LMB relieved the nuclear sequestration of ERK and ADDs and allowed them to re-distribute to the cytoplasm (Fig. 2a-c). These results indicate that ADD2 and ADD3 may translocate into the nucleus, but are quickly transported back to the cytoplasm upon entering the nucleus.

To compare the nuclear exporting potential of the ADD proteins, all three ADD isoforms were fused with the NLS (PKKKRKV) derived from SV40 T-Ag (Fig. 3a), which is imported into the nucleus by its interaction with importin α [30]. Indeed, we found that ADD2 and ADD3 possess much higher capability than ADD1 to counteract the effect of the NLS derived from SV40 T-Ag (Fig. 3b). These results suggest that the discrepancy in the distributions of the ADD isoforms is likely because of differences in their nuclear export capabilities.

### A leucine-rich sequence (^377^FEALMRMLDWLGYRT^391^) in the neck domain is a functional NES for ADD1, but not for ADD2 and ADD3

**Fig. 4:**
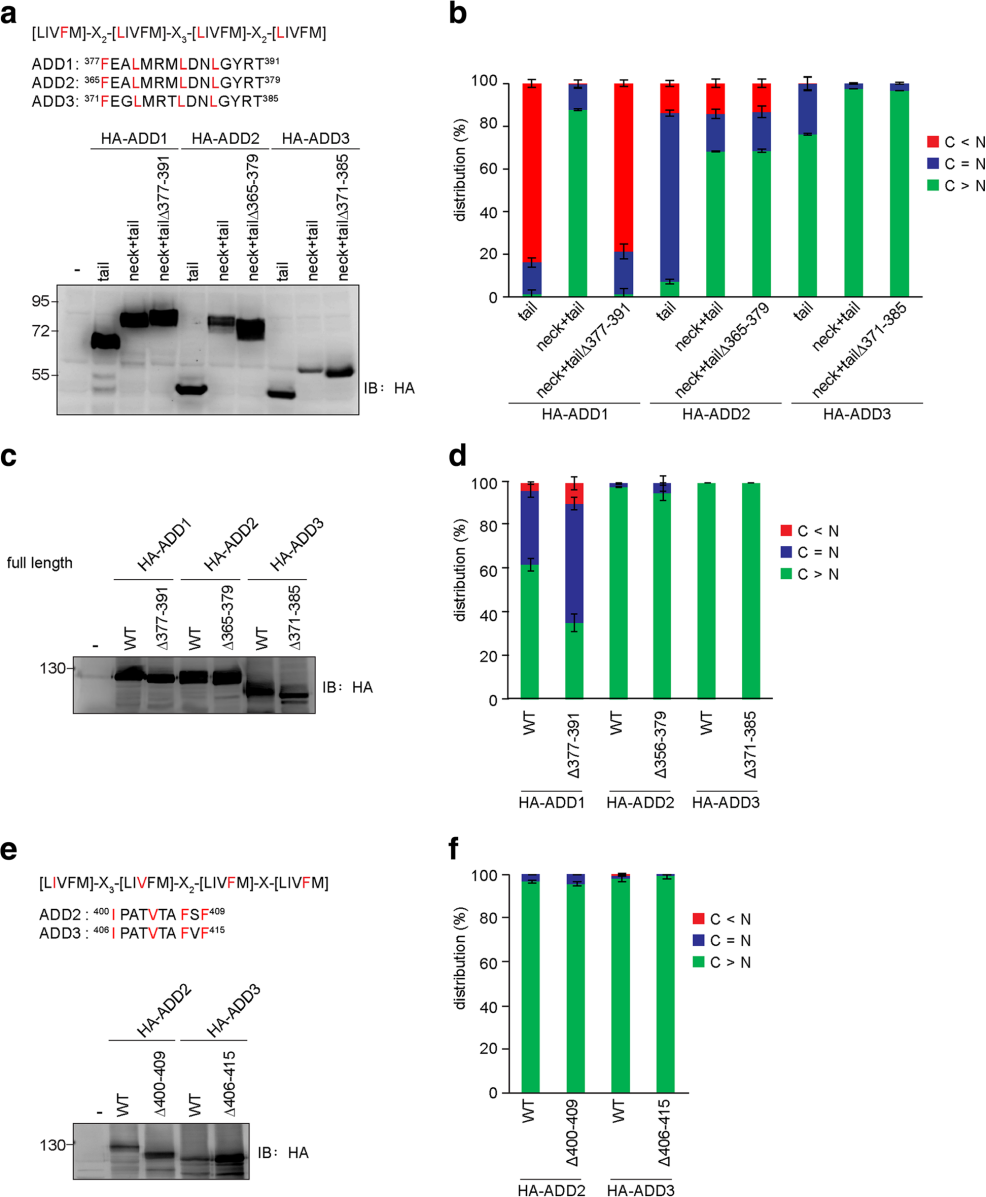
The leucine-rich motif in the neck domain is a functional NES for ADD1, but not for ADD2 and ADD3. **a** A leucine-rich motif (FEALMRMLDWLGYRT) is conserved in all three ADD isoforms. The tail domain alone and the fragment containing both neck and tail domains (neck + tail) with or without the deletion of the leucine-rich motif were constructed. These constructs were transiently expressed in HEK293 cells and their expression was analyzed by immunoblotting with anti-HA antibody. **b** The constructs described in panel a were transiently expressed in NIH3T3 cells and their distribution in the nucleus and cytoplasm was measured (*n* > 300). The values (mean ± SD) are from three experiments. **c** The full-length HA-ADD proteins and their respective mutants with the deletion of the leucine-rich motif (FEALMRMLDWLGYRT) in the neck domain were transiently expressed in HEK293 cells and their expression was analyzed by immunoblotting with anti-HA antibody. **d** The constructs described in panel c were transiently expressed in NIH3T3 cells and their distribution in the nucleus and cytoplasm was measured (*n* > 600). The values (mean ± SD) are from three experiments. **e** The Φ-X_3_-Φ-*X*_2_-Φ-X_1_-Φ motif ("Φ" is a hydrophobic residue) was found in the neck domain of ADD2 and ADD3. HA-ADD2, HA-ADD3, and their respective mutants with the deletion of this motif were transiently expressed in HEK293 cells and their expression was analyzed by immunoblotting with anti-HA antibody. **f** The constructs described in panel e were transiently expressed in NIH3T3 cells and their distribution in the nucleus and cytoplasm was measured (*n* > 600). The values (mean ± SD) are from three experiments

A leucine-rich sequence (FEALMRMLDWLGYRT) that is conserved in the neck domains of all the ADD proteins conforms to the classic NES consensus pattern of [LIVFM]-*X*_2_-[LIVFM]-X_3_-[LIVFM]-*X*_2_-[LIVFM] [9, 10]. We have previously demonstrated that this leucine-rich sequence is a functional NES for ADD1 [21]. In this study, we show that the neck domain of ADD1 counteracted the effect of the NLS in the tail domain (Fig. 4a and b). Deletion of this sequence abolished the nuclear export capability of the neck domain of ADD1 (Fig. 4a and b), indicating that this motif is the only NES in the neck domain of ADD1. Intriguingly, the tail domain of ADD3 by itself was mainly localized in the cytoplasm (Fig. 4b). However, the deletion of this sequence in the neck + tail construct of ADD2 and ADD3 (Fig. 4a and b) or in the full-length ADD2 and ADD3 (Fig. 4c and d) did not increase their distribution in the nucleus, indicating that this leucine-rich sequence is not a functional NES for ADD2 and ADD3.

Our *in-silico* analysis (http://prodata.swmed.edu/LocNES/LocNES.php) [31] predicts that the sequences ^400^IPATVTAFSF^409^ in ADD2 and ^406^IPATVTAFVF^415^ in ADD3 conform to theΦ-X_3_-Φ-*X*_2_-Φ-X_1_-Φ pattern ("Φ" is a hydrophobic residue) for NES. This motif is not present in the neck domain of ADD1. However, the deletion of these two sequences in the neck domains of ADD2 and ADD3 did not increase their distribution in the nucleus (Fig. 4e and f), thus excluding the possibility of these two sequences as NESs.

### ADD proteins may contain multiple NESs in different domains and preferentially bind different exportins

**Fig. 5:**
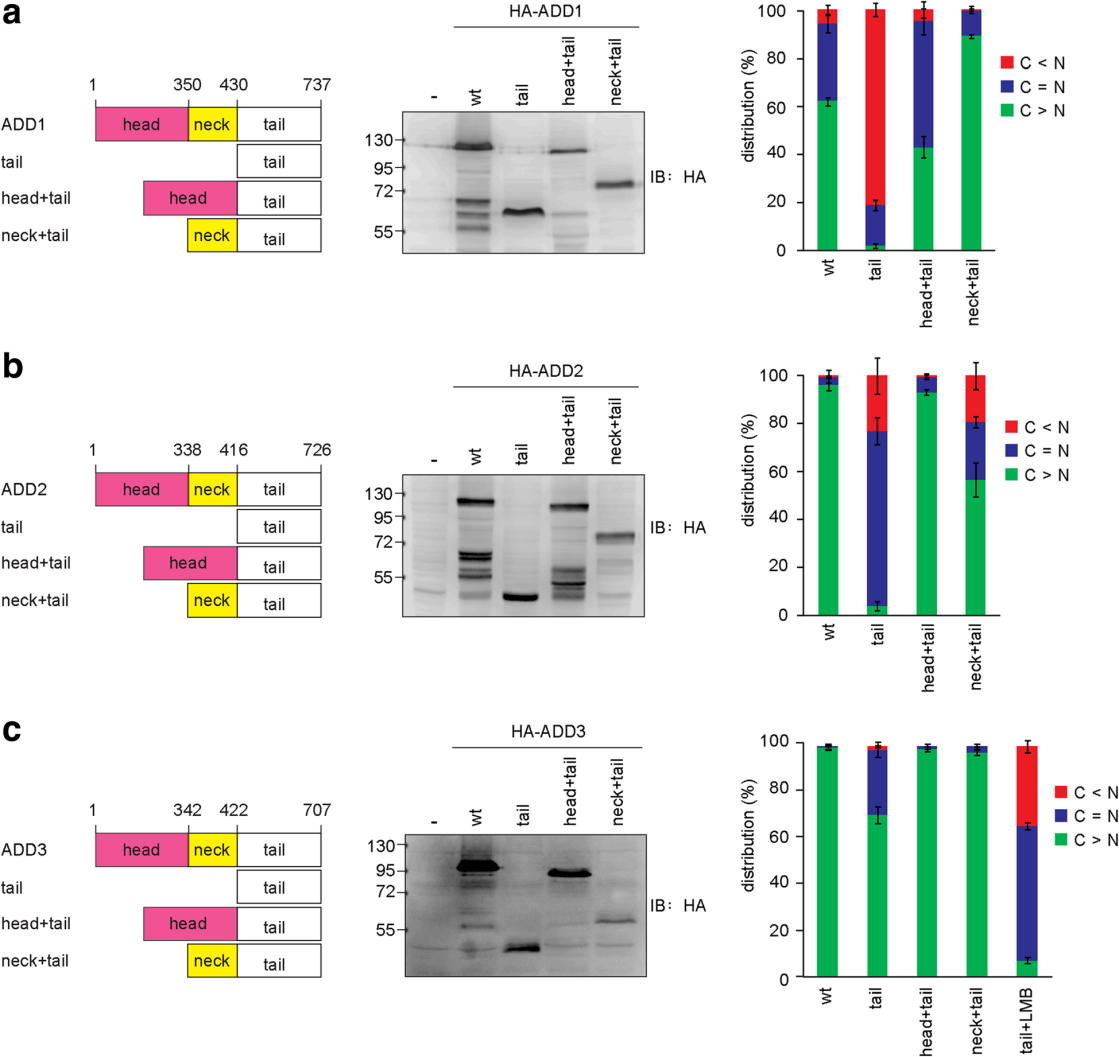
The ADD proteins may contain multiple nuclear export signals in different domains. **a** HA-ADD1, **b** HA-ADD2, **c** HA-ADD3 and their respective constructs for the tail domain (tail) or the tail domain fused to the head (head + tail) or the neck domain (neck + tail) were generated. The constructs were transiently expressed in HEK293 cells and their expression was analyzed by immunoblotting with anti-HA antibody. The constructs were transiently expressed in NIH3T3 cells and their distribution in the nucleus and cytoplasm was measured (*n* > 600). The values (mean ± SD) are from three experiments

**Fig. 6:**
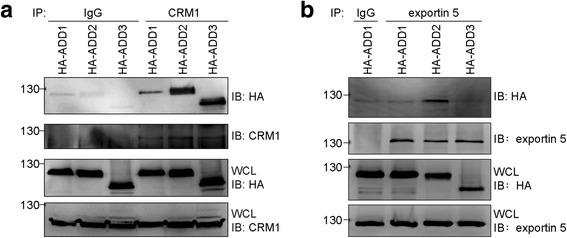
CRM1 interacts with all ADD proteins, whereas exportin5 preferentially interacts with ADD2. **a** HA-ADD1, HA-ADD2, and HA-ADD3 were transiently expressed in HEK293 cells and their expression was analyzed by immunoblotting (IB) with anti-HA antibody. CRM1 was immunoprecipitated (IP) with anti-CRM1 antibody or pre-immune serum (IgG) as the control. The immunocomplexes were analyzed by immunoblotting with anti-HA and anti-CRM1 antibodies. **b** HA-ADD1, HA-ADD2, and HA-ADD3 were transiently expressed in HEK293 cells and their expression was analyzed by immunoblotting (IB) with anti-HA antibody. Exportin5 was immunoprecipitated (IP) with anti-exportin5 antibody or pre-immune serum as the control. The immunocomplexes were analyzed by immunoblotting with anti-HA and anti-exportin5 antibodies. WCL, whole cell lysates

To examine whether the head and neck domains of the ADDs contain functional NESs, the tail domains of ADDs were fused to the head or neck domain. The tail domains of ADD1 and ADD2 were largely localized in the nucleus (Fig. 5a and b) and their head and neck domains were able to counteract the effect of the NLS in their tail domains (Fig. 5a and b), suggesting that the head and neck domains of ADD1 and ADD2 may contain NESs. Interestingly, the tail domain of ADD3 by itself was mainly localized in the cytoplasm (Fig. 5c) and could be sequestered in the nucleus by LMB (Fig. 5c), suggesting that ADD3 may have additional NESs in the tail domain. In addition, we found that CRM1 interacted with all the ADD proteins, whereas exportin5 preferentially interacted with ADD2 (Fig. 6). These results suggest that the ADD proteins may contain multiple NESs in different domains and are preferentially recognized by different exportins.

### Nuclear retention of ADD1 may facilitate its interactions with nuclear proteins

**Fig. 7:**
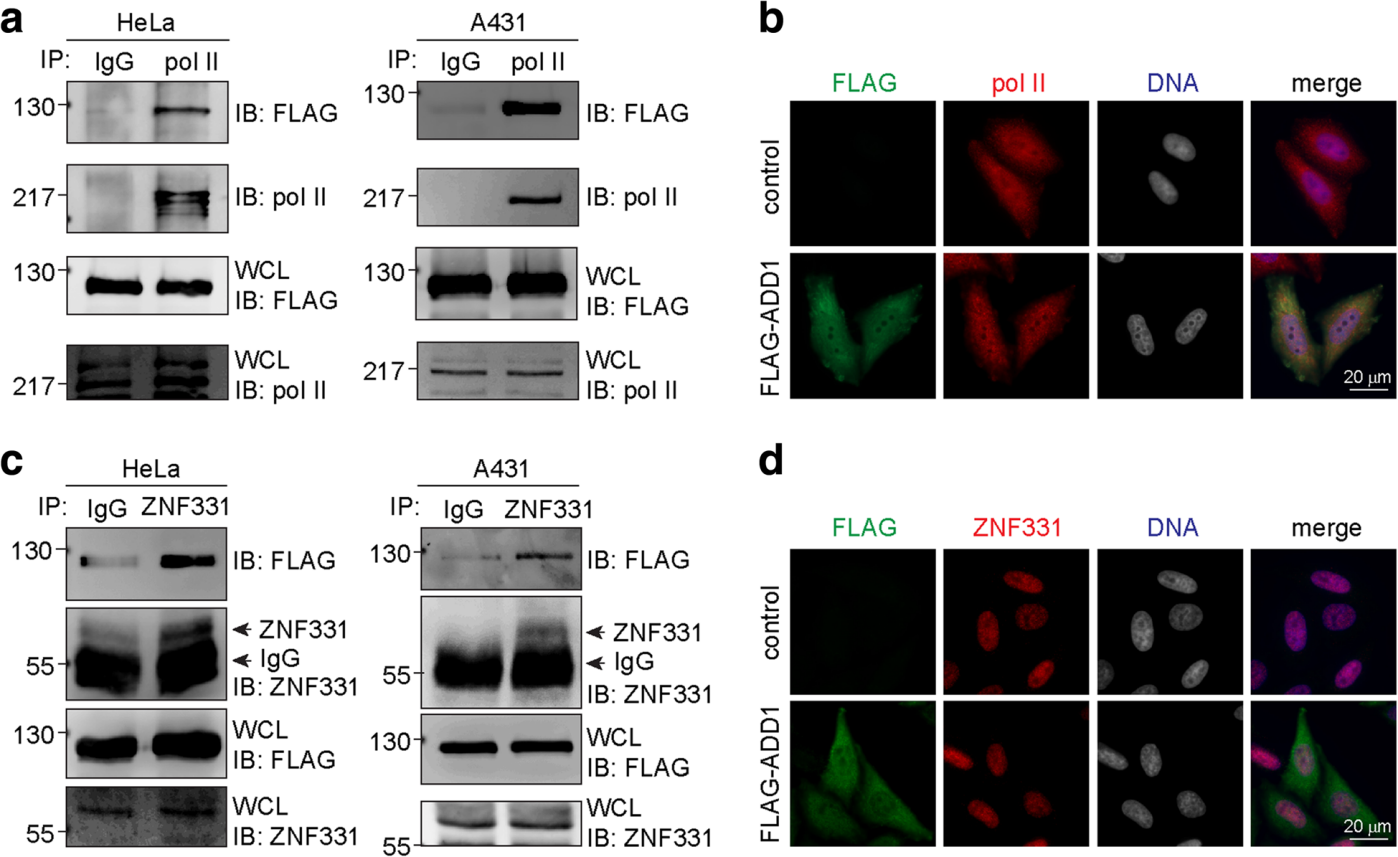
ADD1 interacts with RNA polymerase II and ZNF331. **a** FLAG-ADD1 was stably expressed in HeLa cells and A431 cells. The interaction of FLAG-ADD1 with RNA polymerase II (pol II) was analyzed by co-immunoprecipitation. Pre-immune serum (IgG) was used as the control. Whole cell lysates (WCL) were analyzed by immunoblotting with the indicated antibodies. **b** The HeLa cells stably expressing FLAG-ADD1 were subjected to immunofluorescence staining for FLAG-ADD1, RNA polymerase II, and the nucleus. **c** FLAG-ADD1 was stably expressed in HeLa cells and A431 cells. The interaction of FLAG-ADD1 with ZNF331 was analyzed by co-immunoprecipitation. **d** The HeLa cells stably expressing FLAG-ADD1 were subjected to immunofluorescence staining for FLAG-ADD1, ZNF331, and the nucleus

Compared to ADD2 and ADD3, ADD1 tends to localize in the nucleus (Fig. 1), indicating that ADD1 might have nuclear-specific functions. Indeed, we identified several proteins that interacted with ADD1 by mass spectrometry (data not shown). Among those proteins, the interactions of FLAG-ADD1 with RNA polymerase II and ZNF331 were verified by co-immunoprecipitation in HeLa cells and A431 cells (Fig. 7a and c). In addition, FLAG-ADD1 co-localized with RNA polymerase II and ZNF331 in the nucleus, as visualized by immunofluorescence staining (Fig. 7b and d).

## Discussion

In this report, we characterized the nucleocytoplasmic shuttling of ADDs. All the ADDs were, at least partially, sequestered in the nucleus by LMB (Fig. 2), indicating that the conserved NLS motifs in their tail domains are functional NLSs, through which they are recognized by importin α and transported into the nucleus (Fig. 1b). In addition, we found that ADD2 and ADD3 were much more potent than ADD1 in counteracting the NLS of SV40 T-Ag (Fig. 3). Therefore, the fact that ADD2 and ADD3 are hardly detected in the nucleus with immunofluorescence staining (Fig. 1) is likely due to efficient nuclear export. Moreover, we demonstrated that the conserved leucine-rich sequence in the neck domain is a functional NES for ADD1, but not for ADD2 or ADD3 (Fig. 4). Our results also suggest that the ADDs possess multiple NESs in different domains (Fig. 5). However, our attempts to identify functional NESs with the traditional consensus pattern of Φ1-*X*_2,3_-Φ2-*X*_2,3_-Φ3-X_1_-Φ4 in the ADDs using *in-silico* analysis have not been successful. These results demonstrate that at least some of the NESs in the ADDs may be structure-based rather than sequence-based.

We found that ADD2 and ADD3 were only partially (~40%) sequestered in the nucleus by LMB (Fig. 2), suggesting that the nuclear export of ADD2 and ADD3 might be through both CRM1-dependent and independent mechanisms. In contrast, the nuclear export of ADD1 is mainly CRM1-dependent (Fig. 2). This can at least partly explain why the nuclear export of ADD2 and ADD3 is more efficient than ADD1. Interestingly, we found that ADD2 binds to CRM1 and exportin5 (Fig. 6). In fact, the nuclear export of some proteins has been shown to be dependent on more than one type of exportins. For example, the nuclear export of the complex containing Snail and eEF1a (a protein synthesis elongation factor) is mediated by both CRM1 and exportin5 [32, 33]. In addition, the nuclear export of the thyroid hormone receptor is mediated by CRM1, exportin5, and exportin7 [34].

From the evolution point of view, the ADD genes are possibly duplicated from an ancestor gene due to more sophisticated physiological demands. In addition to their tissue-preference distribution, the ADD isoforms could preferentially respond to different extracellular stimuli. Although ADD2 and ADD3 possess nuclear import capability, the default for ADD2 and ADD3 appears to keep them in the cytoplasm until appropriate extracellular cue. For example, ADD2 was found to accumulate in the nucleus in response to pleiotrophin [26].

In this study, we show that ADD1 interacts with RNA polymerase II and ZNF331 (Fig. 7). These findings suggest that ADD1 may be involved in some nuclear-specific functions. ZNF331 has been considered a transcriptional repressor [35], which functions as a tumor suppressor to suppress the growth and invasion of gastric cancer [36]. In addition, ADD1 was shown to bind and co-localize with RFX1 [37], a nuclear protein that inactivates the transcription of specific genes, such as the microtubule-associated protein MAP1A [38]. Altogether, these data suggest that ADD1 may have a function in transcriptional regulation. On the other hand, the expression of ADD1 is regulated at the transcriptional level by zinc-finger protein 322A (ZNF322A) [39]. ZNF322A is overexpressed in lung cancer patients and is correlated with poor prognosis. It transcriptionally activates downstream genes, such as ADD1 and cyclin D, while suppressing tumor suppressor genes, such as p53 [39]. These data also implicate a potential role of ADD1 in tumorigenesis and/or malignant progression.

Extensive research in the past decade has significantly broadened our view of the role of actin in the nucleus. Nuclear actin plays a key role in transcription, chromatin remodeling, and pre-mRNA processing [40, 41]. In addition, more than 30 actin-binding proteins and new classes of actin-related proteins have been found to be involved nuclear-specific functions, including RNA transcription and processing, nuclear transport, and chromatin remodeling [42]. We have previously demonstrated that ADD1 is localized at the cell-cell junctions and translocated into the nucleus upon loss of cell-cell adhesion [21]. Here, we further demonstrate that ADD1 is capable of interacting with RNA polymerase II and ZNF331. Experiments are in progress to characterize the function of ADD1 in the nucleus.

## Conclusions

In summary, our results demonstrate that ADD family proteins including ADD1, ADD2, and ADD3 possess functional NLS and shuttle between the cytoplasm and nucleus. The preference of ADD2 and ADD3 to localize in the cytoplasm is because of their higher nuclear exporting potential than ADD1. The nuclear retention of ADD1 facilitates its interaction with RNA polymerase II and ZNF331, implicating a potential role for ADD1 in regulating transcription.

## Abbreviations

ADD: Adducin
CRM1: Chromosomal maintenance 1
GFP: Green fluorescent protein
LMB: Leptomycin B
NES: Nuclear export signal
NLS: Nuclear localization signal
